# Personal Health Information Management Among Older Adults: Scoping Review

**DOI:** 10.2196/25236

**Published:** 2021-06-07

**Authors:** Malgorzata Kolotylo-Kulkarni, Deborah E Seale, Cynthia M LeRouge

**Affiliations:** 1 Department of Information Management & Business Analytics College of Business & Public Administration Drake University Des Moines, IA United States; 2 Department of Public Health College of Health Sciences Des Moines University Des Moines, IA United States; 3 Department of Information Systems & Business Analytics College of Business Florida International University Miami, FL United States

**Keywords:** personal health information management, health information management, scoping review, information management, consumer health informatics, medical informatics, patient participation

## Abstract

**Background:**

Older adults face growing health care needs and could potentially benefit from personal health information management (PHIM) and PHIM technology. To ensure effective PHIM and to provide supportive tools, it is crucial to investigate the needs, challenges, processes, and tools used by this subpopulation. The literature on PHIM by older adults, however, remains scattered and has not provided a clear picture of what we know about the elements that play a role in older adults’ PHIM.

**Objective:**

The goal of our review was to provide a comprehensive overview of extant knowledge on PHIM by older adults, establish the status quo of research on this topic, and identify research gaps.

**Methods:**

We carried out a scoping review of the literature from 1998 to 2020, which followed the Preferred Reporting Items for Systematic Reviews and Meta-Analyses extension for Scoping Reviews (PRISMA-ScR) framework. First, we executed a broad and structured search. We then carried out a qualitative analysis of papers pertinent to the topic taking into consideration the five elements of the patient work system as follows: (1) personal-level factors, (2) PHIM tasks, (3) tools used, (4) physical settings of PHIM activities, and (5) socio-organizational aspects.

**Results:**

The review included 22 studies. Consolidated empirical evidence was related to all elements of the patient work system. Multiple personal factors affected PHIM. Various types of personal health information were managed (clinical, patient-generated, and general) and tools were used (electronic, paper-based, and others). Older adults’ PHIM was intertwined with their surroundings, and various individuals participated. The largest body of evidence concerned personal factors, while findings regarding the physical environment of PHIM were scarce. Most research has thus far examined older adults as a single group, and scant attention has been paid to age subgroups.

**Conclusions:**

Opportunities for further PHIM studies remain across all elements of the patient work system in terms of empirical, design science, or review work.

## Introduction

Personal health information management (PHIM) is a process that involves creating, seeking, organizing, and sharing personal health information (PHI) of individuals to be engaged in their lives and their health care [1-5]. Patients who can access and manage their PHI may be more empowered to partner in their care. Effective PHIM can facilitate, for instance, patients’ knowledge of their conditions [6] or adherence to treatment protocols [7]. However, PHIM is often challenging due to, for instance, many sources of information, and although there are tools designed to support PHIM, they differ in their level of accessibility, advancement, and cost.

Health consumers who could greatly benefit from effective PHIM to help support their health care and well-being are older adults. Older adults represent a growing subpopulation with approximately 52.5 million people aged 65 years or older in 2018 (35% increase from 2008) in the United States, and the number is projected to almost double by 2060 [8]. For this population, effective PHIM is of utmost importance as older adults often exhibit high health care needs [9] and costs [10] and may experience a decline in emotional well-being due to their health status [11].

Differences exist among older adults in terms of their experiences related to their health and their health care needs, which may drive different PHIM requirements and digital preferences among subgroups of older adults. These differences often correspond to various age subgroups within the older adult population*.*

For instance, the lives of older adults at midlife are often in flux [12]. They are frequently caretakers within their family dynamics (caring for spouses, children, parents, grandchildren, and/or siblings) and thereby may be managing a large volume of health information. As this group of people move into an older adult phase, they may be working longer or undergoing life transitions, such as retiring, which requires changes in health insurance coverage.

These transitioning older adults may differ from elderly people in their adoption of health technologies [13,14]. Many older adults have multiple health conditions, as comorbidities increase with age [15,16], and older adults with increasing health challenges exhibit high health care utilization [17]. These issues contribute to creating vast amounts of health-related information. Further, the elderly subpopulation is often on a fixed income and must closely manage health care costs. While elderly people may have more time to focus on managing their health information, their health conditions and potential cognitive decline may interfere with their ability to handle PHIM [18].

To ensure effective PHIM, design functional PHIM technology, and enable policymakers to devise practice interventions for older adults, we need to understand older adults’ PHIM practices. The amount of effort and focus that a patient needs to assign to treatment has been coined “patient work” [19]. Such work not only entails the specific activities performed, but also includes and is shaped by the environmental and contextual elements that surround those activities.

Extant research indicates that PHIM is a complex and multidimensional phenomenon, as exemplified in the patient work framework [20]. This framework, while integrating prior models (the work system [21] and the SEIPS model [22]), consolidates the elements that are embodied in or impact patient work as follows: (1) person-related factors, (2) tasks carried out, (3) factors related to the tools used and information managed, (4) characteristics of the physical environment, and (5) socio-organizational aspects [20]. Indeed, to fully understand the landscape of PHIM practices by older adults, research needs to extend beyond one perspective or aspect of PHIM, a single technology, a health condition, or a single group of older adults. Insights go beyond the findings of an isolated study.

While limited PHIM literature reviews that attempt to consolidate extant knowledge on the topic do exist, with each one from a different perspective [4,23-27], few have focused on older adults (a previous study is an example of this research [28]). More so, those reviews did not provide a system view of the various factors that play a role in older adults’ PHIM. Prior reviews examining PHIM by older adults focused on their patient portal use [28], but did not examine older adults’ PHIM practices at a comprehensive general level. Other reviews studied the literature on medication management from the perspective of informal caregivers of older adults [29]. Literature related to older adults thus remains fragmented, and there is a need for an overview of extant empirical evidence on PHIM by older adults, particularly in light of the heterogeneity of PHIM.

The purpose of this review was thus to provide a synopsis of knowledge on PHIM by older adults, determine the status quo of this research, and identify gaps in it. This literature review systematizes and consolidates current empirical evidence on the needs and challenges older adults face, the current PHIM practices they carry out, the tools and information that they use for PHIM, the environment in which they manage their PHI, and the different stakeholders with which they interact. Furthermore, this study explores extant findings in the literature concerning PHIM differences among age subgroups of older adults. In light of the growing importance of electronic PHIM tools, we focused on PHIM literature published in the past two decades.

## Methods

### Overview

Literature reviews are well-recognized for their potential contributions. They have been shown to help establish the status quo of the literature, support theory testing, determine research gaps, and develop theory [30]. Recently, research pointed out the need for more literature review work in the information systems discipline, noted its significance in the field, and proposed suggestions on how rigorous and fruitful reviews may be executed [30].

Scoping reviews are particularly effective in answering broader research questions, carrying out a wider literature search, and providing an overview of research on a given topic [31-35]. They are also useful when examining complex and heterogenous phenomena [35]. To describe research on PHIM by older adults, we have thus carried out a scoping review of the literature on this topic. We were guided by the Preferred Reporting Items for Systematic Reviews and Meta-Analyses extension for Scoping Reviews (PRISMA-ScR) checklist [34].

### Literature Search Strategy

Initially, we carried out several preliminary literature search processes, which enabled us to decide on a set of keywords and databases for the search. Our study’s final literature search process consisted of the following three parts: (1) systematic search using online databases, (2) citation analysis of the full papers found during the search, and (3) citation analysis of four literature reviews published since 2009 related to PHIM. The database search further consisted of the following two stages: (1) the main search using generic PHIM keywords and (2) a detailed search using keywords representing main PHIM tasks identified through initial coding and review of the literature. We also carried out a citation analysis of articles identified during the database search and citation analysis of previous literature reviews by screening the papers cited by those studies (forward citation using Google Scholar). This approach helped us maximize the recall of the articles relevant to the study. We performed the searches between October and November 2019, with an update search conducted in December 2020 and January 2021. The update included Academic Search Complete (replacing Academic Search Premier) and did not include ABI/INFORM due to limited accessibility.

We followed search criteria (Multimedia Appendix 1) established by us for a broad and structured search process to ensure that articles relevant to our research objective and research questions were included. The search criteria were established to balance viability with breadth and comprehensiveness [36]. We focused on research published from 1998 to 2020 to cover the past two decades in order to balance recency (particularly in light of the increasing role of electronic PHIM tools) and comprehensiveness of empirical findings. We began our work by examining research published over two decades and continued to add literature as our work emerged. Due to the nature of the phenomenon (ie, PHIM encompasses multiple elements, such as actors, tools, and technologies), we decided to review only literature that examined PHIM among older adults without a focus on a specific technology (personal health records [PHRs] or wearable devices) or other actors (eg, caregivers).

Furthermore, to ensure the quality of the empirical evidence found and to establish the status of the development of this stream of research, we focused only on papers published in peer-reviewed journals. However, in order to ensure we did not miss any relevant recent findings, which could have been presented at conferences but have not been published in journal outlets, we also looked for conference papers in the 2019-2020 period.

Two researchers determined the articles to be included for the review to warrant their meeting of the inclusion criteria and their cohesiveness. Any ambiguities concerning inclusion were discussed and resolved.

### Analysis of the Literature

To review the literature identified during the search, we carried out a qualitative analysis by adopting coding schema according to the patient work system [20] and using Dedoose. We proposed the patient work framework [20] as a lens from which to organize and connect findings of isolated tasks and tools (technology and others) used by older adults into a system of “patient work.” Carrying out our analysis from the perspective of this framework enabled us to provide a comprehensive and consolidated view of the research on older adults’ PHIM. The lead author did all the coding.

Upon completing the analysis, we summarized (1) the descriptive information about the eligible studies and (2) significant findings extracted from the papers relevant to our research questions.

For the review, we included five papers that also examined the perspectives of older adults’ caregivers. However, we only incorporated findings from older adults’ responses. Discussions of PHIM carried out by caregivers who were also older adults were omitted if the participant’s age was not verifiable. Results not clearly attributed to older adults in the papers were also not included in the review.

We also included papers that examined PHIM by older adults even if they examined younger adults, but only if they also examined subgroups among older adults. We included only findings relevant to older adults and the subgroups among them. This search criterion was included owing to a small number of papers specifically studying older adults aged 50 years or above and carrying out a subgroup analysis.

## Results

### Literature Search Results

As a result of the search, 87 papers were eligible for in-depth examination, and we concluded the search with 22 papers eligible for qualitative analysis. The flowchart indicating the results of the literature search process is presented in Multimedia Appendix 2.

### Study Characteristics

The majority (n=15) of papers were published since 2015. Reviewed research has taken different directions and examined the topic from various perspectives. The papers reviewed were slightly dominated by studies adopting a qualitative approach (13 papers), and eight papers undertook quantitative methods. The studies primarily included interviews, focus groups, survey questionnaires, and other methods such as review of existing patient portals or clinic appointment observations.

Most papers (n=17) examined these topics exclusively from the older adults’ perspective, although five studies also included the point of view of older adults’ informal caregivers.

Most papers (n=16) focused on older adults as a single group and did not distinguish across age subgroups. Details of the six papers focusing on age subgroups are provided in the section Older Adult Subgroup Study Findings.

Overall, concerning the study’s purpose, the papers spanned from PHIM behavior studies and older adults’ views of PHI and use thereof to PHIM technology use (such as patient portals) by older adults.

The summary of key information for the reviewed papers is provided in Multimedia Appendix 3.

### PHIM by Older Adults

Below, we delineate the findings revealed in the literature concerning the elements of the patient work model [20] that play a role in older adults’ PHIM.

#### Person-Related Factors That Drive or Challenge PHIM Among Older Adults

The reviewed literature showed that the major personal factors that drive or challenge older adults’ PHIM span across their background and lifestyle. These factors include attitude toward PHIM [37], demographics [38], health status and behavior [39], literacy [40], lifestyle and quality of life [41], and perceptions of other stakeholders [42]. Many of these elements can vary in their effect on PHIM, as the literature has demonstrated differences and particular complexity when various aspects are studied (or from multiple perspectives). These disparities are exemplified, for instance, in the effects of gender, as some findings have shown that women are more likely to adopt online tools [43], while other findings have indicated that men exhibit more confidence in PHR use [44].

**Figure 1:**
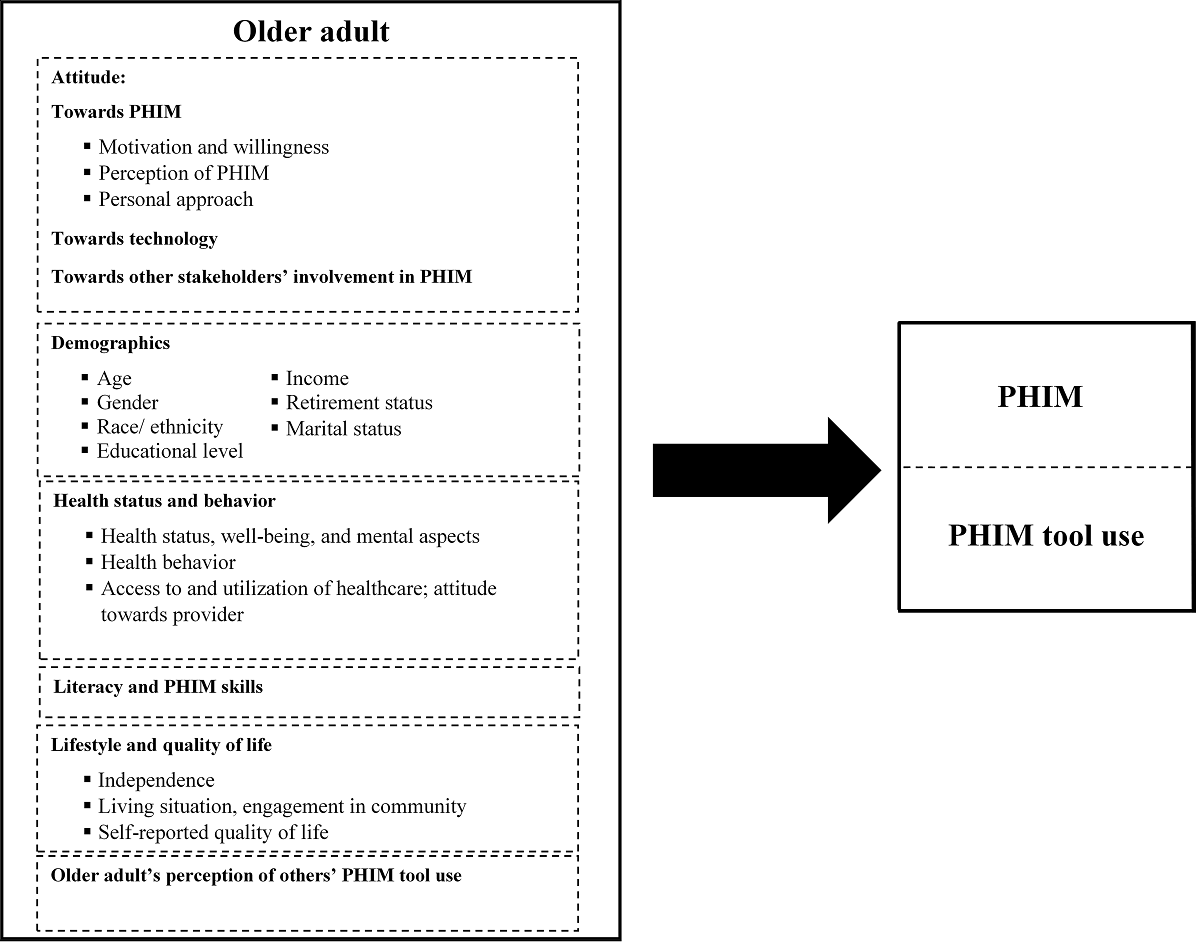
Person-related factors affecting personal health information management (PHIM) and PHIM tool use.

Figure 1 delineates these factors, while Multimedia Appendix 4 provides further details on them.

#### PHIM Tasks Carried Out by Older Adults and Their Characteristics

Managing personal health information involves multiple tasks and is performed over many (not linear) stages.

Older adults search for, collect, or create information [1,37,42,43,45-51]. They also share their PHI with others [1,39,42,43,45-49,51-53], make decisions concerning the storage and management of the information [1,43,45-47,51], and evaluate information [42,43,47,48], for instance, by reconciling conflicting information [47]. Importantly, PHIM tasks also include planning health behaviors with one’s PHI.

Planning health behaviors include medication planning, such as filling pillboxes, purchasing medication, and planning how to keep medication; disposing of old medication; and ordering refills [41,48-50,52,54,55]. Another example is emergency planning, which has been noted as preparing or maintaining information for emergency situations [45,46,51]. These examples of planning as PHIM tasks particularly stand out owing to their predominance in the literature and the contextual nature of PHIM.

**Figure 2:**
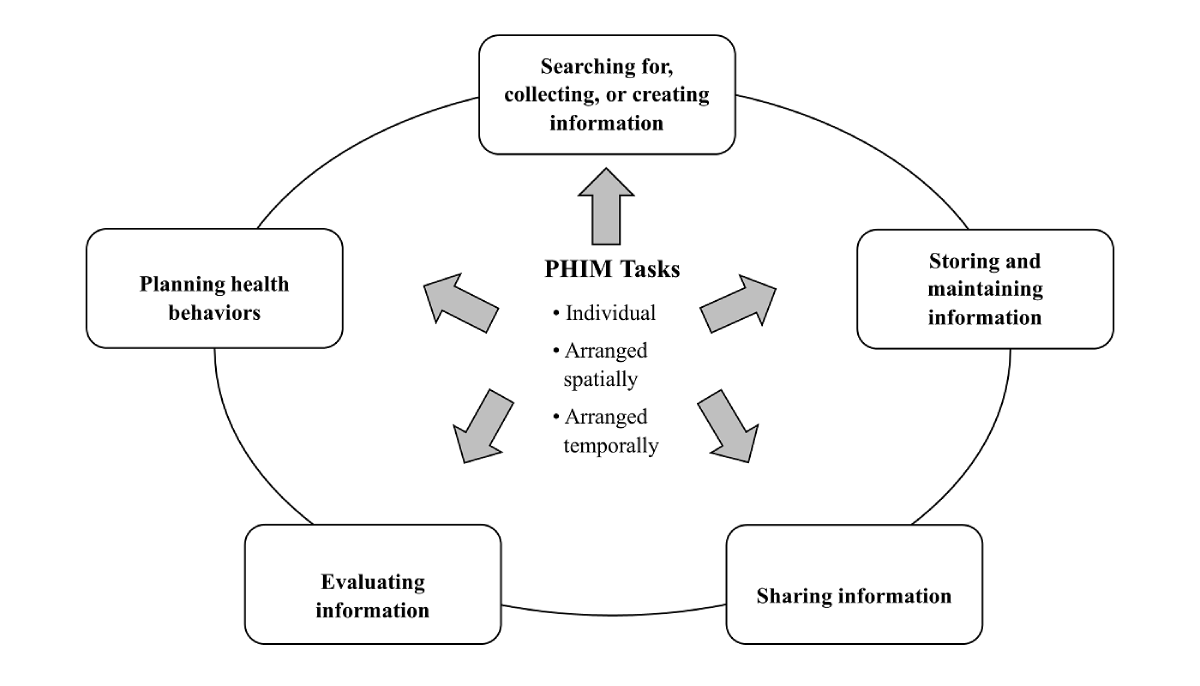
Personal health information management (PHIM) tasks carried out by older adults.

Figure 2 delineates the main PHIM tasks carried out by older adults. Further detailed findings on the tasks are provided in Multimedia Appendix 5 and Multimedia Appendix 6.

Figure 2 also shows the collective nature of PHIM tasks. First, PHIM tasks are highly individual, for instance, to what extent older adults are willing to share their PHI with others [39], and they vary across adults. For example, not everybody engages in various planning health behaviors, such as preparing emergency information [46].

PHIM tasks are also often synergistic with the environment in which they are executed. That is, tasks are intertwined and aligned with the location. For instance, older adults place pillboxes in various visible locations around the house to serve as reminders to take medication as a planned health behavior or choose to store their PHI record where it was originally generated (such as where blood pressure measurement is taken) [48]. Some older adults may choose to keep their nonprescription and prescription medication lists separate when reconciling differences between the two medication types [47].

Lastly, tasks are also temporally arranged, that is, tasks are entwined with one’s routine and other life activities. For instance, older adults may create information by checking their weight as part of their morning routine [48].

#### Personal Health Information Managed by Older Adults and the Types of Solutions or Tools That They Use to Support Their PHIM

Older adults manage various types of personal health information spanning clinical data [1,37,42,43,46,47,49], such as lab results [1,42,46,49]; patient-generated health data that includes clinical information [1,42,43,46-48,50,51,56], such as self-care logs [1,43,48,51], and information related to logistics and administration [1,42,46,51], such as emergency contact information [46,51]; and general health and wellness data, such as online information on medication side effects [42,47] and health educational materials [51]. Detailed findings on the information that older adults manage are shown in Multimedia Appendix 7.

Tools, solutions, and technologies that are currently offered or which older adults use to manage their PHI include electronic approaches [1,37,40,42,43,46-49,51,52], such as computers or laptops [42,46,51] and the internet [37,40,42-44,47-49,51,52]; paper-based approaches [1,37,41,42,46-48,51,54], such as printouts [37,42] and calendars [51,54]; and medical, every day, and other objects that include tangible objects [41,46-48,50,51,54,55], such as portable file cabinets [47] and pill boxes [41,48,50,51,54,55], and intangible objects [1,41,51], such as memory [1,41,51].

Detailed findings on the tools and methods older adults use for PHIM are provided in Multimedia Appendix 8 and Multimedia Appendix 9.

#### Physical Environments That Older Adults Occupy During PHIM and Their Characteristics

PHIM activities that are carried out by older adults occur in one’s house [1,46,55] and away from home [1,48]. Older adults use multiple locations in their homes for PHIM purposes, such as posting PHI on the back of their front door or fridge door [1,46]. PHIM also crosses boundaries, as older adults, for instance, keep PHI at hand and carry it around (such as in their wallets) [46].

**Figure 3:**
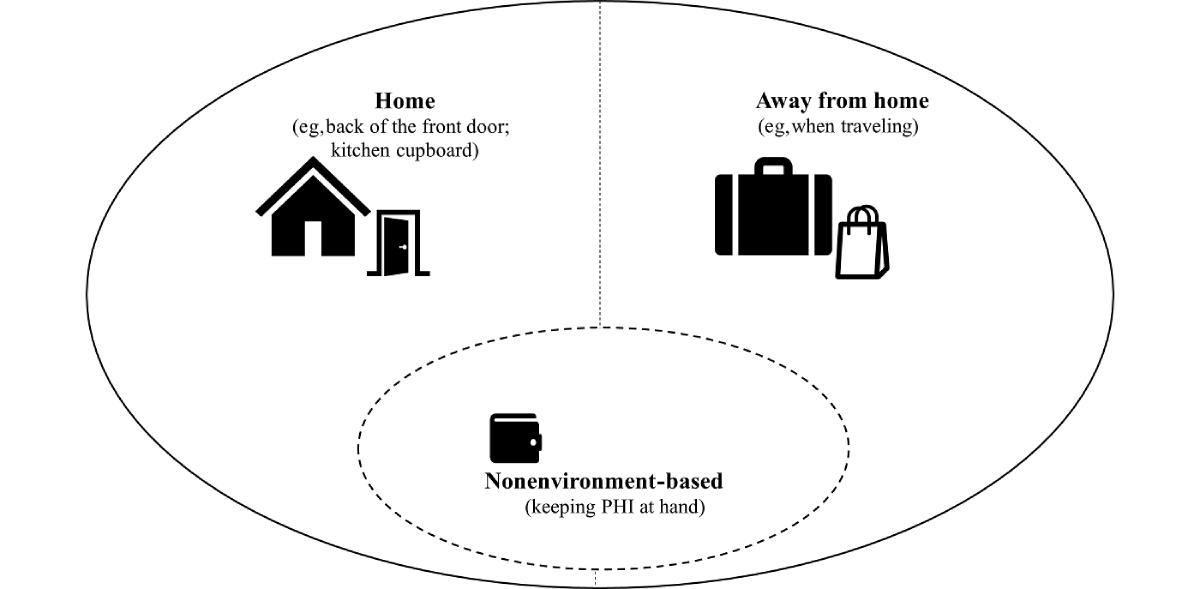
Physical environment of personal health information management by older adults. PHI: personal health information.

Figure 3 presents the physical environment of older adults’ PHIM, and Multimedia Appendix 10 provides detailed findings in the literature on this aspect.

#### Socio-Organizational Environment in PHIM Among Older Adults: Stakeholders Involved

Many people are involved in older adults’ PHIM and collaborate with them in different capacities to manage their PHI. These stakeholders include persons in the older adult’s immediate circle (personal relationships), such as family, friends, and neighbors [1,37,39,41-44,46,47,51,53-56], and health care workers or retirement community staff, such as health care providers and professionals [1,37,42,43,46-48,50,56]. Sometimes, older adults particularly seek the help of their friends or relatives who have medical knowledge or expertise [42].

**Figure 4:**
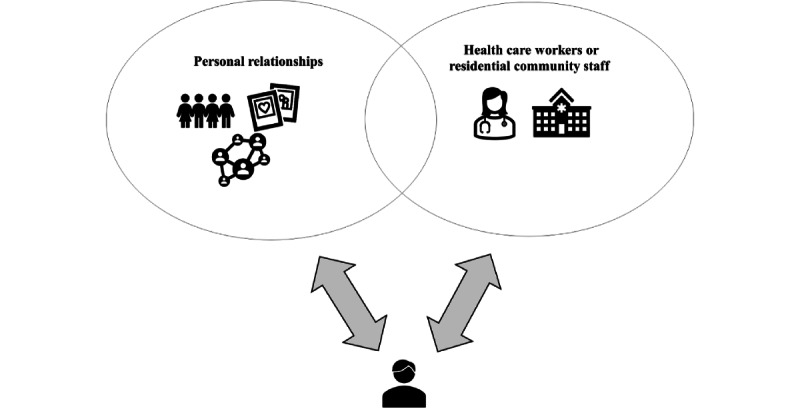
Socio-organizational environment of older adult's personal health information management: stakeholders involved.

Figure 4 demonstrates the stakeholders with whom older adults interact during PHIM, and Multimedia Appendix 11 and Multimedia Appendix 12 delineate detailed literature findings on them.

### Older Adult Subgroup Study Findings

Table 1 presents a summary describing the papers that carried out an analysis based on age subgroups.

Six of the PHIM studies investigated older adult age subgroups (person factor) (Table 1). One examined a single older adult subgroup [41] and five compared two or more older adult subgroups [37,40,44,51,52]. As distinguishing among age subgroups was not their primary focus, two studies only reported one finding each related to age subgroup differences [40,51].

The number of older adult subgroups (person factor) studied and the age ranges of subgroups varied (5-year and 10-year increments, generational, median split, and very old) across the six studies (Table 1). Nevertheless, the findings were relatively consistent for the youngest and eldest of older adults regarding PHIM tasks, tools, and socio-organizational environmental factors. Physical environmental factors were notably absent from age subgroup findings.

A medication study [41] and one of the medical record studies [37] found that the eldest of older adults perceived the effective management of clinical PHI tasks to be necessary for (1) remaining in their homes [41], (2) communicating with their providers [37], and (3) taking better care of their health [37]. At the same time, all but one [40] of the four medical record studies found that the eldest of older adults were the least likely to use digital records and the least prepared to manage clinical PHI using digital technologies [37,44,52].

The eldest of older adults were also more likely to perceive the need for assistance from stakeholders and tools (digital and nondigital). The eldest subgroups shared their medical records to allow others to participate in their care [37] and relied on personal and health community caregivers to help them plan PHIM [51] and manage PHIM tools, that is, pill dispensers [41] and digital health records [52].

In contrast, the youngest of older adults were more likely to use and be prepared to use digital records [37,44,52], but less likely to use medical records to involve the family in their care [37] and more likely to use medical records to care for their children [37]. The findings are mixed for the two studies that examined middle older adult subgroups [37,52].

**Table 1:** Older adult personal health information management studies with age subgroup findings.

Authors, year published	PHIM^a^ focus	Data collection		Sample size	Number of subgroups	Age subgroups
		Period	Source			
Arcury et al, 2017 [40]	ePortal^b^	2014-16	Interview	200	4	55-59, 60-64, 65-69, and ≥70 years
Gordon & Hornbrook, 2016 [52]	ePortal	2013-14	Admin; Survey	231,084; 3660^c^	3	65-69, 70-74, and 75-79 years
Huvila et al, 2018 [37]	PHIM & paper medical record.	2012	Survey	354	3	<52, 52-66, and ≥67 years
Logue & Effken, 2012 [44]	ePHR^d^	~2009^e^	Survey	38	2	65-77 and 78-93 years
Turner et al, 2021^f^ [51]	PHIM	5-year period^g^	Interview; survey	88; 38^h^	4	60-69, 70-79, 80-89, and 90-99 years
Westerbotn et al, 2008 [41]	PHIM & medication management	2005	Interview	25	1	85-97 years

^a^PHIM: personal health information management.

^b^ePortal: electronic portal.

^c^Administrative data from the patient ePortal used to determine portal use (n=231,084) and identify a sample for the survey (n=3660).

^d^ePHR: electronic personal health record.

^e^Data collection period unspecified. It was inferred from a sentence in the manuscript.

^f^Turner et al, 2021 was published online in 2020.

^g^Exact timeframe unspecified.

^h^Subset of interview participants (n=88) willing to be contacted for the feedback survey (n=38).

## Discussion

### Implications of the Study

Research at large has recognized the peculiarities of midlife in terms of physical health, cognitive function, and social role [12]. Accordingly, scholars recognize the disparities between older adults at midlife and elderly people in terms of information behavior (eg, health information seeking [57]). However, the literature that we reviewed has largely not considered these differences. Only six papers carried out age subgroup analyses and only one paper included in the review examined the differences in the PHIM practices of midlife and elderly older adult subgroups.

Furthermore, the studies that recognized differences across age subgroups among older adults (eg, older adults and elderly people) adopted various cutoff ages among the subgroups. The ambiguity in the cutoff age used to distinguish older adults and elderly people in the reviewed studies suggests that there is no generally accepted cutoff age. Lack of a clear cutoff age for these two subgroups challenges a systematic approach to research on these two groups.

While an absolute cutoff age for older population subgroups creates some challenge, we need to look to the nature and purpose of the study for a path forward and to connect the literature. Underlying much of the PHIM literature that recognizes different subgroups for an older population is recognition that people typically have different generational idiosyncrasies as well as health needs at different stages of life. Belonging to a given generation can, conceivably, affect the socio-cultural characteristics of health consumers, thus potentially influencing their practices and approaches to PHIM technology. For example, research has acknowledged the uniqueness of age subgroups among older adults. Specifically, the literature has recognized that older adults at midlife are at a pivotal time in their life; hence, they have been referred to as pivotal agers [58]. In our review, we sought an objective means to consider subgroups among older adults; hence, we assumed the cutoff as the retirement age. However, various factors (generation, life experiences, etc) could be considered here. Extant literature has shown various approaches, with some research, for instance, driving the split by year of birth [37].

Our review corroborates the role that the various patient work elements of the PHIM system play for health consumers, as has been suggested in prior research [20]. The patient work model [20] has been shown to be valuable in observing the factors from different life and environment areas. It is particularly useful here in drawing more attention to socio-organizational aspects that affect older adults’ PHIM. PHIM is affected by and intertwined with one’s personal life as well as physical and socio-organizational environment. Thus, the factors involved in or influencing PHIM should be considered together to create a system, especially for those older adults who have comorbidities, and should be customized to an adult’s unique health status.

Our review also shows that older adults adopt a variety of tools to support their PHIM, whereby not only electronic but also paper-based solutions are still commonly used.

Extant research has shown the role of the various elements of the patient work model [20]. However, the elements have received differing levels of attention. Our findings indicate that most empirical evidence in the literature thus far concerns person-related factors and the least evidence pertains to the physical environment of PHIM.

The complex and multidimensional nature of PHIM caused the nature of the search process to be quite challenging. Studies were found in multiple academic domains, and it was difficult to obtain a holistic perspective of which papers should be included and excluded. Our evaluations of whether studies should be included in the review were somewhat ambiguous and challenging, and necessitated establishing clear and detailed inclusion and exclusion criteria. Similar difficulties have been reported previously [28]. Moreover, the lack of existence of PHIM as a Medical Subject Headings (MeSH) term and the inconsistent use of keywords across papers complicated the discovery process.

Our review adds to the extant PHIM research. Our investigation extends prior work, which discussed the challenges of PHIM [23]. Our review also adds to previous literature reviews on PHIM tools [25,28], by examining the various types of PHIM tools used by older adults and the information they manage. We also extended the findings of earlier work [29] by corroborating the role that caregivers and other stakeholders play in older adults’ PHIM. We extended the results of prior work [4], which also examined PHIM through the patient work model [20]. Our review included findings through 2020 and took a distinct perspective by focusing on older adults. Our findings are also consistent with research on medication management by older adults, which also used the patient work framework perspective [59].

Our review showcases several potential avenues for future empirical or design science research related to the various patient work elements that play a role in older adults’ PHIM. Further research is needed to examine the idiosyncratic characteristics and challenges of older adults at midlife and elderly people. Additionally, it would be valuable to extend this research by investigating specific PHIM tools and tailoring their design toward different age subgroups among older adults. Furthermore, scant evidence regarding the characteristics of PHIM tasks and PHIM location suggests the need to inspect the nature (ie, attributes) of PHIM activities carried out by older adults and the physical environment of such activities.

PHIM research can also be extended by examining, for instance, the nature of involvement of the socio-organizational environment in older adults’ PHIM practices. For example, this may be accomplished by focusing on the viewpoint of other stakeholders involved in older adults’ PHIM, such as caregivers and providers.

### Limitations

The limitations of our review’s findings pertain primarily to the possibility of omitting relevant papers and the limited scope of the findings presented.

First, limiting our review to research published in peer-reviewed journals over the last 22 years and conference proceedings published in the last 2 years could have resulted in omission of relevant findings.

During the search, we did not include keywords such as those reflecting all the different types of PHIM technologies (eg, activity monitoring), as our focus was on older adults’ characteristics and PHIM practices. It is conceivable, though, that literature on specific PHIM tools, which were omitted this way, could have also included empirical evidence on older adults’ PHIM. It is thus possible that not every tool type was discovered in our review.

Lastly, the challenges of paper identification (caused by, for instance, the complexity of the topic and the occurrence of publications in many areas, as delineated above) could have resulted in erroneous omission of papers.

### Conclusions

This paper contributes to research by consolidating and systematizing fragmented evidence from the literature on PHIM by older adults and establishing the status quo of research in this area. Our review shows that older adults’ PHIM constitutes a system of patient work. Extant literature on this topic has so far focused largely on the personal characteristics of older adults, and the least attention has been paid to the physical environment of their PHIM. Most of the reviewed research did not differentiated between midlife and elderly people. Additionally, our review suggests that this area of research is still fairly recent.

Our review may be valuable for practitioners. Policymakers, for instance, may take into account the personal factors and older adults’ socio-organizational environment affecting PHIM identified in our review to potentially pinpoint areas that necessitate or could be facilitated by practice interventions or organizational support. Furthermore, policymakers may also consider the use of paper-based and electronic tools by older adults in the context of information blocking and patient portal utilization.

The findings of our review may also encourage developers to consider the individual elements of the PHIM system in design and acknowledge the interdependencies among them. Such recognition could make the design of PHIM tools, such as patient portals, more holistic, resulting in tools that support PHIM as a system.

## Appendix

Multimedia Appendix 1 Parameters of the literature search process.

Multimedia Appendix 2 Preferred Reporting Items for Systematic Reviews and Meta-Analyses (PRISMA) diagram of the literature search process.

Multimedia Appendix 3 Personal health information management study summary.

Multimedia Appendix 4 Person-related aspects that play a role in personal health information management by older adults.

Multimedia Appendix 5 Personal health information management tasks carried out by older adults.

Multimedia Appendix 6 Characteristics of personal health information management tasks carried out by older adults.

Multimedia Appendix 7 Personal health information managed by older adults.

Multimedia Appendix 8 Tools used by older adults for the purposes of personal health information management.

Multimedia Appendix 9 Differences in personal health information management tools used across age subgroups among older adults.

Multimedia Appendix 10 Physical environment of personal health information management by older adults.

Multimedia Appendix 11 Stakeholders involved or who play a role in older adults' personal health information management.

Multimedia Appendix 12 Differences in the socio-organizational environment in personal health information management across age subgroups among older adults.

## Abbreviations

PHI: personal health information
PHIM: personal health information management
PHR: personal health record
