# Supplementary material for: Personal Health Information Management Among Older Adults: Scoping Review
Source: J Med Internet Res. 2021 Jun 7;23(6):e25236. doi: 10.2196/25236 (PMC8218209; doi:10.2196/25236)
Supplement: Multimedia Appendix 1 [file jmir_v23i6e25236_app1.docx]

**Multimedia Appendix 1.** Parameters of the literature search process.

| Parameter |  |  |
| --- | --- | --- |
|  |  |  |
| **Databases queried** | ABI/INFORM Collection, Academic Search Premier, Academic Search Complete, CINAHL (Ebsco), JSTOR, Medline Proquest, PubMed, ScienceDirect (Elsevier), WebofScience, |  |
| **1^st^ Database Search** |  |  |
| **Keywords** | Health information management | AND older adults |
|  | “health information management” OR “personal health information management” OR “consumer health information management” OR “manage health personal health information” OR “managing personal health information” | “older adults” OR “older patients” OR elderly |
| **MESH Terms** | Health Information Management | Adult OR Frail Elderly |
| PubMed |  |  |
| Medline Proquest |  |  |
| CINAHL (Ebsco) | Health Information Management/ Evaluation/ Methods/ Psycho-social Factors/ Utilization | Middle Age OR Aged+ |
| **2^nd^ Database Search** |  |  |
| **Keywords** | Health information management | AND older adults |
|  | "health information seeking" OR "health information search" OR " health information use" OR " health information tracking" OR "health information integration" OR "health information collection" OR "health information sharing" OR “health information storage” OR “health information creation” OR “health information retrieval” OR “health information check” OR “activity monitoring” OR “medication management” | “older adults” OR “older patients” OR elderly |
| **Selection criteria** | 1. Studies examining PHIM by older adults 50 + in the US and abroad with respect to:    1. personal characteristics which drive, impact, or challenge their PHIM    2. PHIM practices and techniques    3. physical settings of PHIM and where PHIM tools are used, and the characteristics of those settings    4. the type of information managed and tools to support their PHIM and to handle challenges of PHIM    5. the social - organizational environment in PHIM 2. Studies whose sample includes subgroups of various ages if the age groups are delineated by range and if the study includes subgroups among older adults. 3. Studies that were published between Jan 1998 and Dec 2020. 4. Peer-reviewed journal articles. 5. Keywords appearing only in titles or abstracts. |  |
| **Exclusion criteria** | 1. Studies including multiple age groups, except for studies explicitly examining differences between subgroups among older adults. 2. Studies with sample age delineated only by mean or median. 3. Studies examining only the perspective of other stakeholders. 4. Studies focusing on PHIM technology, desired features for it, or evaluations of PHIM tools with little findings on older adults’ abilities or practices or with insufficient focus on older adults. 5. Not written in English. 6. Conference proceedings, dissertations, books; journal abstracts. 7. Journal articles: commentaries, editorials, letters to the editor, conceptual articles, reports, methodology papers, reviews, meta-analyses. 8. Records for which full text was not available. |  |
