# Supplementary material for: Personal Health Information Management Among Older Adults: Scoping Review
Source: J Med Internet Res. 2021 Jun 7;23(6):e25236. doi: 10.2196/25236 (PMC8218209; doi:10.2196/25236)
Supplement: Multimedia Appendix 2 [file jmir_v23i6e25236_app2.docx]

## Multimedia Appendix 2. Preferred Reporting Items for Systematic Reviews and Meta-Analyses (PRISMA) diagram of the literature search process.

Additional records identified through citation analysis

**(n = 9)**

Records identified through database searching
**(n = 2535)

1^st^ search: n = 333** (ABI/INFORM Collection = 45; Academic Search Premier = 6; CINAHL = 91; Academic Search Ultimate = 0; JSTOR = 0; Medline ProQuest = 14; PubMed = 166; ScienceDirect (Elsevier) = 7; WebofScience = 4)

**2^nd^ search: n = 2202** ABI Inform Collection = 180; Academic Search Premier = 361; Academic Search Ultimate = 138; CINAHL = 461; JSTOR = 2; Medline ProQuest = 112; PubMed = 551; ScienceDirect (Elsevier) = 294; WebofScience = 103)

## Identification

## Screening

Records excluded

**(n = 826)**

Full-text articles assessed for eligibility

**(n = 105)**

Records screened

**(n = 931)**

Records after duplicates removed

**(n = 931)**

Full-text articles excluded, with reasons
**(n = 83)**

(58 = irrelevant topic or insufficient focus;
5 = perspective of caregivers;
8 = not journal publications;
5 = wrong age group or age not provided; 3 = not empirical;
2 = small sample of older adults; 2 = not available)

## Eligibility

## Included

Studies included in qualitative synthesis

**(n = 22)**
