# Supplementary material for: Personal Health Information Management Among Older Adults: Scoping Review
Source: J Med Internet Res. 2021 Jun 7;23(6):e25236. doi: 10.2196/25236 (PMC8218209; doi:10.2196/25236)
Supplement: Multimedia Appendix 3 [file jmir_v23i6e25236_app3.docx]

## **Multimedia Appendix 3.** Personal health information management study summary.

| Authors  [Reference #] | Pub  Year^1^ | Study Purpose | Target Population/ Sample Age | PHIM  Focus^2^ | Subgroup Analysis | Methodology | Analysis^3^ |
| --- | --- | --- | --- | --- | --- | --- | --- |
|  |  |  |  |  |  |  |  |
| Arcury et al. | 2017 | To identify modifiable factors influencing the use of patient portals by older adults receiving care at providers for low income and ethnically diverse consumers. To examine the association between patient portal utilization with perceived usefulness and usability. | Adults 55 and above | Med Rec (ePortal) | Yes | Interview | Quant |
| Crotty et al. | 2015 | To examine how older adults and their caregivers view and approach the sharing of older adults’ PHI. | Adults 75 and older; caregivers of older adults | PHIM (Sharing) | No | Focus Group | Qual |
| Francis et al. | 2006 | To examine partnerships between older adults and their caregivers with regard to medication management. | Adults 60-106; caregivers (29-91) | PHIM (Medication) | No | Interview | Qual |
| Gordon & Hornbrook | 2016 | To examine differences across older adults’ use of and access to patient portals depending on their age, race, and ethnicity. | Adults 65-69; 70-74; 75-79 | Med Rec (ePortal) | Yes | Multimethod | Quant |
| Hartzler et al. | 2018 | To examine older adults’ views and use of personal health information. | Adults 60 and older | PHIM (Aging) | No | Focus Group | Qual |
| Haverhals et al. | 2011 | To examine the challenges which older adults and their caregivers face when managing medication. | Adults 73-90; caregivers (age 48-57) of older adults | PHIM (Medication) | No | Multimethod | Qual |
| Huvila et al. | 2018 | To assess the preferences and motivations of older adults ordering health records. | Adults ≤50; 51-66; 67 and older | Med Rec (Paper & Digital) | Yes | Survey | Quant |
| Kim & Choi | 2019 | To examine the role of socio-demographic factors, health, and attitude towards technology in older adults’ willingness to share PHI. | Adults 61 - 94 | PHIM (Sharing) | No | Interview | Quant |
| Kim & Fadem | 2018 | To investigate older adults’ attitudes toward, perception of usability, and requirements for a patient portal. | Adults 65 and older | Med Rec (ePortal) | No | Multimethod | Mixed |
| Lakey et al. | 2009 | To examine older adults’ preferences, use of, and knowledge of medication management tools. | Adults 73-98 | PHIM (Medication) | No | Multimethod | Quant |
| Logue & Effken | 2012 | To explore factors that influence older adults’ adoption of PHRs. | Adults 65-93 | Med Rec (ePHR) | Yes | Survey | Quant |
| Mickelson et al. | 2015 | To examine older adults’ approaches to the use of cognitive artifacts in the management of medication. | Adults 65-86 | PHIM (Medication) | No | Multimethod | Mixed |
| Portz et al. | 2019 | To examine and describe user interface, user experience, intent to use, and use behavior of a patient portal among older adults with multiple chronic conditions. | Adults 65+ | Med Rec (ePortal) | No | Focus Group | Qual |
| Roux et al. | 2019 | To explore older adults’ medication management and the spatial aspect of it. | Adults 68-90; caregivers of older adults | PHIM (Medication) | No | Multimethod | Qual |
| Swanlund | 2010 | Older adults’ experiences of facilitators and challenges of their medication management. | Adults 75-97 | PHIM (Medication) | No | Interview | Qual |
| Taha et al. | 2014 | To examine effects of Internet experience,  numeracy, and education on older adults’ ability to perform tasks using a patient portal. | Adults 60-85 | Med Rec (ePortal) | No | Multimethod (Usability) | Quant |
| Tomlinson et al. | 2020 | To examine how older adults and their carers experience post-discharge medication management. | Adults 75 and above; caregivers of older adults | PHIM (Medication) | No | Interview | Qual |
| Turner et al. | 2018 | Older adults’ accounts of how they manage their PHI. | Adults 60-98 | PHIM (Emergency) | No | Multimethod | Qual |
| Turner et al. | 2019 | To explore PHIM practices carried out by older adults. | Adults 60 years and older | PHIM (Aging) | No | Multimethod | Qual |
| Turner et al. | 2021^4^ | To explore PHIM needs and practices of older adults. | Adults 60 and above | PHIM (Aging) | Yes | Multimethod | Qual & Quant |
| Westerbotn et al. | 2008 | To examine how older adults experience their medication management regimen. | Adults 85 or above | PHIM (Medication) | Yes | Interview | Qual |
| Zettel-Watson & Tsukerman | 2016 | To investigate older adults’ view and usage of online health management tools. | Adults 50-87 | Online Hlth Tech | No | Survey | Quant |
| ^1^ Pub Year = Publication Year  ^2^ PHIM Focus (Med Rec = Medical Record, ePHR = Patient-controlled electronic personal health record, ePortal = clinically-controlled, patient-accessible electronic personal health record, Online Hlth Tech = Online Health Technologies, PHIM = Personal Health Information Management)  ^3^ Quant = Quantitative, Qual = Qualitative  ^4^ Turner et al. 2021 was published online in 2020. | | | | | | | |
