# Supplementary material for: Personal Health Information Management Among Older Adults: Scoping Review
Source: J Med Internet Res. 2021 Jun 7;23(6):e25236. doi: 10.2196/25236 (PMC8218209; doi:10.2196/25236)
Supplement: Multimedia Appendix 4 [file jmir_v23i6e25236_app4.docx]

## Multimedia Appendix 4. Person-related aspects that play a role in personal health information management by older adults.

| Personal aspects of older adults affecting PHIM | Key factors | Key highlights | References |
| --- | --- | --- | --- |
|  |  |  |  |
| **Attitude** |  |  |  |
| **Attitude towards PHIM** |  |  |  |
|  | Motivation and willingness for PHIM and PHIM tool use | Motivations include finding believing one *should* do it, that it is necessary to participate in healthcare, to gain an overview of health condition, to keep track of health status or prevent illness, to understand information, to get informed, general interest | (Huvila et al., 2018; Turner et al., 2018; Turner et al., 2020) |
|  |  | Some are proactive about staying independent and managing their medication | (Swanlund, 2010) |
|  |  | Older adults’ willingness to share information differs for different stakeholders | (T. K. Kim & Choi, 2019) |
|  |  | Beliefs in PHR impact on health goals are related to intention to use PHR | (Logue & Effken, 2012) |
|  |  | Some do not want to be reminded of aging | (Turner et al., 2018) |
|  |  | Intention to use PHR is positively related to the likelihood of accepting incentives to use it | (Logue & Effken, 2012) |
|  |  | Those who see the benefit of PHR over other methods are more likely to be motivated by incentives | (Logue & Effken, 2012) |
|  |  | Incentives motivation is positively related with one’s confidence in using PHR | (Logue & Effken, 2012) |
|  | Perception of PHIM | Older adults find PHIM overwhelming | (Haverhals et al., 2011) |
|  |  | Older adults don’t like the look of posted information | (Turner et al., 2018) |
|  |  | Older adults do not see PHIM as necessary, don’t have a need for PHIM tool, or PHIM is carried out by another stakeholder | (Turner et al., 2018; Zettel-Watson & Tsukerman, 2016) |
|  | Personal approach | Persons procrastinate about PHIM | (Turner et al., 2018) |
|  |  | Older adults who are confident about using PHR is related to their interest in trying it | (Logue & Effken, 2012) |
| **Attitude towards technology** |  |  |  |
|  |  | Those with a positive attitude toward technology are more willing to share their information with developers | (T. K. Kim & Choi, 2019) |
|  |  | Trust of information varies across older adults | (Mickelson et al., 2015) |
|  |  | Fear of technology use leads to difficulties using the tool (e.g. handling upgrades) | (Portz et al., 2019) |
|  |  | Older adults are concerned over lost information, privacy, potential for their identity stolen and see those as barriers | (Zettel-Watson & Tsukerman, 2016) |
| **Attitude towards others’ involvement in PHIM** |  | Older adults’ preferences for caregiving and sharing PHI with caregivers vary; some find it unnerving when caregivers take the lead in medication management | (Hartzler et al., 2018; Roux et al., 2019) |
|  |  | Some older adults are not willing to involve family in their care and share their PHI | (Crotty et al., 2015) |
|  |  | Some older adults distrust others e.g. when managing medication | (Westerbotn et al., 2008) |
|  |  | Older adults feel alone with challenges of managing medication if they receive no help | (Westerbotn et al., 2008) |
|  |  | Older adults don’t ask for help with medication as they don’t want to feel like a burden | (Westerbotn et al., 2008) |
| **Demographics** |  |  |  |
|  | Age | Older age is related to greater willingness to share PHI | (T. K. Kim & Choi, 2019; Zettel-Watson & Tsukerman, 2016) |
|  |  | Older adults 66 and above are more inclined to involve family members in their care | (Huvila et al., 2018) |
|  |  | Age is related to different emergency planning | (Turner et al., 2018) |
|  |  | Younger older adults (less than 77) report they know what health resources are available online | (Logue & Effken, 2012) |
|  |  | Adults age 51-66 are less confident about understanding health information than adults 66 and above | (Huvila et al., 2018) |
|  |  | Older seniors (77 and above) report less confidence in their ability to use online PHRs | (Logue & Effken, 2012) |
|  |  | Older adults 70-79 less likely to be able to carry out PHIM with patient portal | (Gordon & Hornbrook, 2016) |
|  |  | Older age is related to less use of online health management tools | (Zettel-Watson & Tsukerman, 2016) |
|  |  | Older adults 80 and above are more likely to involve others in emergency planning and to take up a filing approach to PHIM | (Turner et al, 2020) |
|  | Gender | Women are less likely to share PHI with the government | (T. K. Kim & Choi, 2019) |
|  |  | Women are more likely to adopt online tools | (Zettel-Watson & Tsukerman, 2016) |
|  |  | Women are more likely to be impacted by family to use PHRs | (Logue & Effken, 2012) |
|  |  | Men are more likely to see PHRs with their healthcare needs | (Logue & Effken, 2012) |
|  |  | Men show more confidence in using PHRs | (Logue & Effken, 2012) |
|  | Race/ ethnicity | More White older adults use the patient portal than non-White^[[1]](#footnote-1),^^[[2]](#footnote-2)^; non-Whites prefer non-online tools for some PHIM tasks^[[3]](#footnote-3)^ and some have lower perceived ability to perform PHIM task using PHIM tool^[[4]](#footnote-4)^ | (Arcury et al., 2017; Gordon & Hornbrook, 2016) |
|  |  | Older Hispanic women are more likely to be influenced by family members to use PHRs | (Logue & Effken, 2012) |
|  | Educational level | Higher education negatively impacts willingness to share PHI; College education positively affects PHIM tool use; Education above high school is related to portal use | (Arcury et al., 2017; Gordon & Hornbrook, 2016; T. K. Kim & Choi, 2019) |
|  | Income | Higher income is related to patient portal use | (Arcury et al., 2017) |
|  | Retirement status | Retired vs. not retired is related to different emergency planning types (plan by self, by others, or no planning) | (Turner et al., 2018) |
|  | Marital status | Being married is associated with patient portal use | (Arcury et al., 2017) |
| **Health status and behavior** | Health status, well-being, & mental aspects | Older adults see sharing PHI as more crucial during emergencies/ declined health status | (Crotty et al., 2015) |
|  |  | Lower health status is related to greater patient portal use^[[5]](#footnote-5)^; Decreased motor ability and sensory alertness are seen to hinder medication management | (Arcury et al., 2017; Swanlund, 2010) |
|  |  | Older adults are unable to manage PHI if it is beyond their cognitive abilities, clear-headedness supports managing medication; Decreased mental capacity or memory is seen as a hindrance to managing medication or remembering login or password to PHIM tool; | (Haverhals et al., 2011; Swanlund, 2010; Westerbotn et al., 2008) |
|  |  | Decreased cognitive abilities drive differing perspectives among older adults and caregivers | (Francis et al., 2006) |
|  |  | Temporally not feeling well hinders managing medication | (Swanlund, 2010) |
|  | Health behavior | Older adults engage in activities to maintain their mental, physical, emotional health, and independence | (Hartzler et al., 2018) |
|  | Access to and utilization of healthcare; attitude towards provider | Having multiple providers is related to PHR use intention, to belief PHR fits one’s healthcare needs | (Logue & Effken, 2012) |
|  |  | Receiving care in multiple locations is related to perceived PHR benefits and the use of PHR | (Logue & Effken, 2012) |
|  |  | Having easier access to healthcare is related perceiving the benefits of PHR and use of it | (Logue & Effken, 2012) |
|  |  | Receiving care at an urban (rather than rural) clinic is associated with patient portal use | (Arcury et al., 2017) |
|  |  | Concern over the amount of medication drives strategies to reduce it | (Haverhals et al., 2011) |
|  |  | Older adults’ preference to work with provider is related to perceiving the benefits of PHR and their use of PHR | (Logue & Effken, 2012) |
| **Literacy and PHIM skills** |  | eHealth literacy is related to emergency planning by self or no planning vs. by others; related to patient portal/ PHR use, believing one has resources to use PHR and that it fits with one’s healthcare needs | (Arcury et al., 2017; Logue & Effken, 2012; Turner et al., 2018) |
|  |  | Low digital health literacy is associated with involving others in emergency planning, proxy sharing, and passive seeking | (Turner et al. 2020) |
|  |  | Health literacy is related to patient portal use | (Arcury et al., 2017) |
|  |  | Numeracy is associated with older adults’ ability to perform health management tasks using a portal | (Taha et al., 2014) |
|  |  | Older adults need knowledge to fully understand their medication | (Swanlund, 2010) |
|  |  | Awareness of the need for information helps medication management | (Swanlund, 2010) |
|  |  | Self-efficacy is related to the perception of having all resources needed to use PHR | (Logue & Effken, 2012) |
|  |  | Motivation to learn new things is related to believing PHR use would yield desired goals | (Logue & Effken, 2012) |
|  |  | Older adults who can use the Internet are more willing to perform PHIM online or able to perform health tasks using a portal | (Gordon & Hornbrook, 2016; Taha et al., 2014) |
|  |  | Older adults confident about communicating online or are at comfort with technology are more likely to use PHR | (Arcury et al., 2017; Logue & Effken, 2012) |
|  |  | Older adults who know what health resources are available online are more likely to be motivated by incentives to use PHR | (Logue & Effken, 2012) |
|  |  | Older adults who know how to find health resources online are more likely to be interested in trying a PHR | (Logue & Effken, 2012) |
|  |  | Using email and frequency of Internet use are related to patient portal use | (Arcury et al., 2017) |
|  |  | Older adults are not willing to use the PHIM tool if they feel they don’t have PHIM skills (e.g. ability to interpret PHI) | (S. Kim & Fadem, 2018) |
|  |  | Some older adults misunderstand information | (Mickelson et al., 2015) |
|  |  | Older adult is not aware of PHIM tool or PHIM tool feature’s existence | (Portz et al., 2019; Zettel-Watson & Tsukerman, 2016) |
|  |  | Lack of portal experience is related to the use of traditional means of communication | (S. Kim & Fadem, 2018) |
|  |  | Using computer is associated with active seeking and to take up 1) an independent but sharing and 2) tossing approach to PHIM | (Turner et al., 2020) |
|  |  | Using portals is related to active seeking | (Turner et al., 2020) |
| **Lifestyle and quality of life** | Independence | Older adults self-sufficient about managing medication see it as natural to continue | (Westerbotn et al., 2008) |
|  | Living situation, engagement in community | Older adults living independently vs. assisted living or retirement homes are less likely to carry out emergency planning by others | (Turner et al., 2018) |
|  |  | Living only with spouse is related to portal use | (Arcury et al., 2017) |
|  | Self-reported quality of life | Higher quality of life is associated with the use of online health management tools | (Zettel-Watson & Tsukerman, 2016) |
| **Older adult’s perception of others’ PHIM tool use** |  |  |  |
|  |  | Perception of whether and how the provider will use the PHIM tool and whether they would want the person to use it affects older adult’s decision to use it (patient portal) | (S. Kim & Fadem, 2018) |
|  |  | Older adults interested in observing others use PHR are also more confident in using PHR | (Logue & Effken, 2012) |

1. Detailed findings on the differences across ethnic groups for usage of patient portal for PHIM tasks may be found in original source. [↑](#footnote-ref-1)
2. Ethnicity was found insignificant in multivariate analysis in Arcury et al. (2017) [↑](#footnote-ref-2)
3. Preference for non-digital modalities is not significantly different between White older adults and Black, Filipino, Latino, and Chinese for all PHIM tasks. [↑](#footnote-ref-3)
4. Detailed findings on the differences across ethnic groups for usage of patient portal for PHIM tasks may be found in original source. [↑](#footnote-ref-4)
5. Charlson index was found insignificant in multivariate analysis in Arcury et al. (2017) [↑](#footnote-ref-5)
