# Supplementary material for: Personal Health Information Management Among Older Adults: Scoping Review
Source: J Med Internet Res. 2021 Jun 7;23(6):e25236. doi: 10.2196/25236 (PMC8218209; doi:10.2196/25236)
Supplement: Multimedia Appendix 5 [file jmir_v23i6e25236_app5.docx]

## Multimedia Appendix 5. Personal health information management tasks carried out by older adults.

| PHIM process tasks | Key highlights on tasks and activities carried out | References |
| --- | --- | --- |
|  |  |  |
| **Searching for, collecting, or creating information** | Creating information, e.g. creating documents, scheduling an appointment | (S. Kim & Fadem, 2018; Portz et al., 2019; Turner et al., 2018; Turner et al., 2020; Zettel-Watson & Tsukerman, 2016) |
|  | Searching for information, advice, explanation of information managed | (Hartzler et al., 2018; Huvila et al., 2018; S. Kim & Fadem, 2018; Swanlund, 2010; Turner et al., 2019; Turner et al., 2020) |
|  | Accessing, collecting, or tracking information, ordering a copy of record | (Hartzler et al., 2018; Haverhals et al., 2011; Huvila et al., 2018; S. Kim & Fadem, 2018; Mickelson et al., 2015; Turner et al., 2018; Turner et al., 2019; Turner et al., 2020; Zettel-Watson & Tsukerman, 2016) |
| **Sharing information** | Sharing or communicating information | (Crotty et al., 2015; Francis et al., 2006; Gordon & Hornbrook, 2016; Hartzler et al., 2018; S. Kim & Fadem, 2018; Mickelson et al., 2015; Portz et al., 2019; Turner et al., 2018; Turner et al., 2019; Turner et al., 2020; Zettel-Watson & Tsukerman, 2016) |
|  | Making PHI available for use for others | (Turner et al., 2018) |
|  | Coordinating information across multiple service providers | (Hartzler et al., 2018; Haverhals et al., 2011; S. Kim & Fadem, 2018) |
| **Storing & maintaining information** | Creating lists or one’s own forms | (Turner et al., 2018) |
|  | Overseeing administration of PHI; keeping it up to date; organizing it (e.g. alphabetically); handling new PHI | (Hartzler et al., 2018; Haverhals et al., 2011; Turner et al., 2018; Turner et al., 2019; Turner et al., 2020; Zettel-Watson & Tsukerman, 2016) |
|  | Making or keeping copies or printing document | (Haverhals et al., 2011; Zettel-Watson & Tsukerman, 2016) |
| **Evaluating information** | Reconciling discrepancies, revising information | (Haverhals et al., 2011; Mickelson et al., 2015) |
|  | Checking information (e.g. lab results) | (S. Kim & Fadem, 2018; Zettel-Watson & Tsukerman, 2016) |
|  | Interpreting information | (Mickelson et al., 2015) |
| **Planning health behaviors** | Medication planning (e.g. filling pillboxes, purchasing, planning how to keep medication; disposing of old medication; ordering refills) | (Gordon & Hornbrook, 2016; Lakey et al., 2009; Mickelson et al., 2015; Portz et al., 2019; Roux et al., 2019; Swanlund, 2010; Westerbotn et al., 2008) |
|  | Emergency planning | (Turner et al., 2018; Turner et al., 2019; Turner et al., 2020) |
