# Supplementary material for: Personal Health Information Management Among Older Adults: Scoping Review
Source: J Med Internet Res. 2021 Jun 7;23(6):e25236. doi: 10.2196/25236 (PMC8218209; doi:10.2196/25236)
Supplement: Multimedia Appendix 6 [file jmir_v23i6e25236_app6.docx]

## Multimedia Appendix 6. Characteristics of personal health information management tasks carried out by older adults.

| Characteristics of PHIM tasks | Key highlights | References |
| --- | --- | --- |
|  |  |  |
| **Individuality** | The way that tasks are carried out, their frequency, and preferences for them (e.g. extent of sharing) vary across people | (Crotty et al., 2015; Francis et al., 2006; Mickelson et al., 2015; Tomlinson et al., 2020; Turner et al., 2018; Turner et al., 2019) |
|  | Arranged per one’s skills and limitations | (Mickelson et al., 2015; Swanlund, 2010) |
| **Arranged spatially** | PHIM tasks are arranged around one’s location (e.g. around the house in ways that facilitate those tasks and help overcome challenges) | (Haverhals et al., 2011; Mickelson et al., 2015; Roux et al., 2019; Swanlund, 2010) |
| **Arranged temporally** | PHIM is arranged around one’s daily routines (e.g. medication is taken after breakfast) | (Haverhals et al., 2011; Mickelson et al., 2015; Roux et al., 2019; Swanlund, 2010; Tomlinson et al., 2020) |
