# Supplementary material for: Personal Health Information Management Among Older Adults: Scoping Review
Source: J Med Internet Res. 2021 Jun 7;23(6):e25236. doi: 10.2196/25236 (PMC8218209; doi:10.2196/25236)
Supplement: Multimedia Appendix 7 [file jmir_v23i6e25236_app7.docx]

## Multimedia Appendix 7. Personal health information managed by older adults.

| Types of PHI managed | Key highlights and artifacts | References |
| --- | --- | --- |
|  |  |  |
| **Clinical** | Medical procedures | (Turner et al., 2018) |
|  | Medical records | (Huvila et al., 2018) |
|  | Lab/test results | (Hartzler et al., 2018; S.; Portz et al., 2019; Turner et al., 2018) |
|  | Lab results including treatment options for non-normal values | (S. Kim & Fadem, 2018) |
|  | Medication-related information (instructions, effects, side effects, interactions) | (Haverhals et al., 2011; S. Kim & Fadem, 2018) |
| **Patient-Generated Health Data - CLINICAL** | Everyday health data (e.g. cholesterol, blood pressure, stress, fitness, diet), self-care logs | (Hartzler et al., 2018; Mickelson et al., 2015; Turner et al., 2020; Zettel-Watson & Tsukerman, 2016) |
|  | Family medical history | (Turner et al., 2018) |
|  | Condition(s), allergies | (Turner et al., 2018; Turner et al., 2020) |
|  | Medication-related information (list, notes, questions, requests, photos) | (Hartzler et al., 2018; S. Kim & Fadem, 2018; Mickelson et al., 2015; Swanlund, 2010; Tomlinson et al, 2020; Turner et al., 2018; Turner et al., 2020) |
|  | Nonprescription-related medication information including vitamins and supplements (list, use) | (Haverhals et al., 2011) |
| **Patient-Generated Health Data – Logistics & Administrative** | End-of-life information (e.g. DNR status) | (Turner et al., 2018; Turner et al., 2020) |
|  | Emergency contact information | (Turner et al., 2018; Turner et al., 2020) |
|  | Scheduling or provider contact information | (Hartzler et al., 2018; S. Kim & Fadem, 2018; Turner et al., 2018) |
|  | Insurance coverage (and related) information | (Turner et al., 2018; Turner et al., 2020) |
|  | Religious beliefs | (Turner et al., 2018) |
|  | Contact information or date of birth | (Turner et al., 2018) |
|  | Provider preference | (Turner et al., 2018) |
| **General health & wellness** | Medication-related information (side effects) | (Haverhals et al., 2011; S. Kim & Fadem, 2018) |
|  | Health education materials | (Turner et al., 2020) |
