# Supplementary material for: Personal Health Information Management Among Older Adults: Scoping Review
Source: J Med Internet Res. 2021 Jun 7;23(6):e25236. doi: 10.2196/25236 (PMC8218209; doi:10.2196/25236)
Supplement: Multimedia Appendix 8 [file jmir_v23i6e25236_app8.docx]

## Multimedia Appendix 8. Tools used by older adults for the purposes of personal health information management.

| PHIM tools used | Key artifacts | References |
| --- | --- | --- |
|  |  |  |
| **Electronic** | Computer/ hard drive, laptop | (S. Kim & Fadem, 2018; Turner et al., 2018; Turner et al., 2020) |
|  | Internet (online websites, search engines, email); social media or Web 2.0; PHRs / patient portals | (Arcury et al., 2017; Gordon & Hornbrook, 2016; Haverhals et al., 2011; Huvila et al., 2018; S. Kim & Fadem, 2018; Logue & Effken, 2012; Mickelson et al., 2015; Portz et al., 2019; Turner et al., 2020; Zettel-Watson & Tsukerman, 2016) |
|  | Smartphone / phone | (Gordon & Hornbrook, 2016; S. Kim & Fadem, 2018; Portz et al., 2019; Turner et al., 2018; Turner et al., 2020) |
|  | Secure messaging or text messaging | (Gordon & Hornbrook, 2016; Hartzler et al., 2018) |
|  | Memory stick | (Turner et al., 2018) |
| **Paper-based** | Print-outs or copies | (Huvila et al., 2018; S. Kim & Fadem, 2018) |
|  | Paper forms (from provider or self-made), logs, records, lists, checklists, charts, or post-its | (Hartzler et al., 2018; Haverhals et al., 2011; Mickelson et al., 2015; Turner et al., 2018; Turner et al., 2020; Westerbotn et al., 2008) |
|  | Calendars | (Lakey et al., 2009; Turner et al., 2020) |
| **Medical, everyday, and other objects: Tangible objects** | Medical and emergency communication devices (e. g. emergency necklace) | (Mickelson et al., 2015; Turner et al., 2018; Turner et al., 2020) |
|  | Other – equipment and everyday objects (e. g. weight scale, bags; paper bag; envelope, wallet or purse; watch) | (Lakey et al., 2009; Mickelson et al., 2015; Roux et al., 2019; Turner et al., 2018) |
|  | Portable file cabinet | (Haverhals et al., 2011) |
|  | Medication – related (pill boxes, dispensers, medi-sets; vials; medication package inserts) | (Lakey et al., 2009; Mickelson et al., 2015; Roux et al., 2019; Swanlund, 2010; Turner et al., 2018; Turner et al., 2020; Westerbotn et al., 2008) |
| **Intangible objects** | Person’s own memory | (Hartzler et al., 2018; Westerbotn et al., 2008; Turner et al., 2020) |
