# Supplementary material for: Personal Health Information Management Among Older Adults: Scoping Review
Source: J Med Internet Res. 2021 Jun 7;23(6):e25236. doi: 10.2196/25236 (PMC8218209; doi:10.2196/25236)
Supplement: Multimedia Appendix 9 [file jmir_v23i6e25236_app9.docx]

## Multimedia Appendix 9. Differences in personal health information management tools used across age subgroups among older adults.

| Differences across subpopulations in terms of PHIM tools | Key highlights | References |
| --- | --- | --- |
|  |  |  |
| **Differences in PHIM tools used** | Older adults 70-79 less likely to use the patient portal, register an account | (Gordon & Hornbrook, 2016) |
|  | Older adults 70-74 or 75-79 tend to prefer non-online means of PHIM than older adults 65-69^[[1]](#footnote-1)^ | (Gordon & Hornbrook, 2016) |
|  | Elderly (older than 66) are less interested than older adults (51-66) in reading their medical record online | (Huvila et al., 2018) |

1. Significant differences for preferences for non-digital modalities across the 3 age group 65-69 vs. 70-74 vs. 75-79 vary for different PHIM tasks. [↑](#footnote-ref-1)
