# Supplementary material for: Personal Health Information Management Among Older Adults: Scoping Review
Source: J Med Internet Res. 2021 Jun 7;23(6):e25236. doi: 10.2196/25236 (PMC8218209; doi:10.2196/25236)
Supplement: Multimedia Appendix 10 [file jmir_v23i6e25236_app10.docx]

## Multimedia Appendix 10. Physical environment of personal health information management by older adults.

| Location of PHIM | Key highlights | References |
| --- | --- | --- |
|  |  |  |
| **Home** | Older adults carry out PHIM in various locations at home or utilize multiple points at home to support it (back of the front door, fridge door, kitchen cupboard, kitchen table, work surface, bathroom sink, table in living room) | (Hartzler et al., 2018; Roux et al., 2019; Turner et al., 2018) |
| **Away from home** | Older adults keep medication with them when traveling | (Mickelson et al., 2015) |
|  | Older adults keep track of miles they walk, for instance, around the neighborhood | (Hartzler et al., 2018) |
| **Non-environment - based** | Older adults keep PHI close at hand | (Turner et al., 2018) |
