# Supplementary material for: Personal Health Information Management Among Older Adults: Scoping Review
Source: J Med Internet Res. 2021 Jun 7;23(6):e25236. doi: 10.2196/25236 (PMC8218209; doi:10.2196/25236)
Supplement: Multimedia Appendix 11 [file jmir_v23i6e25236_app11.docx]

## Multimedia Appendix 11. Stakeholders involved or who play a role in older adults' personal health information management.

| Stakeholders involved in or who play a role in older adults’ PHIM | Key highlights on stakeholders | References |
| --- | --- | --- |
|  |  |  |
| **Older adult’s relationships** | Family, friends, neighbors; (includes those with medical knowledge or expertise) | (Crotty et al., 2015; Francis et al., 2006; Hartzler et al., 2018; Haverhals et al., 2011; Huvila et al., 2018; S. Kim & Fadem, 2018; Lakey et al., 2009; Logue & Effken, 2012; Roux et al., 2019; Tomlinson et al., 2020; Turner et al., 2018; Turner et al., 2020; Westerbotn et al., 2008; Zettel-Watson & Tsukerman, 2016) |
| **Healthcare workers or residential community staff** | Healthcare providers, professionals | (Hartzler et al., 2018; Haverhals et al., 2011; Huvila et al., 2018; S. Kim & Fadem, 2018; Mickelson et al., 2015; Swanlund, 2010; Tomlinson et al., 2020; Turner et al., 2018; Zettel-Watson & Tsukerman, 2016) |
|  | Pharmacy/ pharmacist | (Haverhals et al., 2011; Mickelson et al., 2015; Swanlund, 2010; Tomlinson et al., 2020; Zettel-Watson & Tsukerman, 2016) |
|  | Professional caregivers | (Roux et al., 2019; Westerbotn et al., 2008) |
|  | Residential or retirement community staff | (Hartzler et al., 2018; Turner et al., 2018) |
