# Supplementary material for: Personal Health Information Management Among Older Adults: Scoping Review
Source: J Med Internet Res. 2021 Jun 7;23(6):e25236. doi: 10.2196/25236 (PMC8218209; doi:10.2196/25236)
Supplement: Multimedia Appendix 12 [file jmir_v23i6e25236_app12.docx]

## Multimedia Appendix 12. Differences in the socio-organizational environment in personal health information management across age subgroups among older adults.

| Differences across subpopulations in the role of stakeholders | Key highlights | References |
| --- | --- | --- |
|  |  |  |
| Differences in stakeholders involved across age groups | Older adults 66 and above are more willing to discuss their medical records with healthcare professional | (Huvila et al., 2018) |
|  | Younger seniors (less than 77) are more influenced by family members than their physician to use PHRs | (Logue & Effken, 2012) |
